# Supplementary material for: Genetic canalization of nutrient resorption in the widespread grass Phragmites australis under salt stress
Source: eLife. 2026 Oct 26;15:RP111792. doi: 10.7554/eLife.111792 (PMC13707377; doi:10.7554/eLife.111792)
Supplement: Supplementary file 2 [file elife-111792-supp1.docx]

Supplementary File 1

Table A The lineages of *Phragmites australis* in the common garden.

| Lineage/Ecotype | Lineage description | Sample Size |
| --- | --- | --- |
| AU | Genotypes with haplotype P from Oceania, often named FEAU in the previous papers | 7 |
| CN_E | Genotypes mostly with haplotype P mainly from the Eastern China | 38 |
| CN_N | Genotypes mostly with haplotypes M and O mainly from the Northern China | 37 |
| EU | Genotypes mostly with haplotype M from Europe and Africa | 10 |
| EU_NA | Invasive genotype with haplotype M in the North America | 14 |
| NA | Native genotypes from the North America | 4 |
| Freshwater wetlands | Genotypes originating from non-saline freshwater habitats across China, including the shores of inland rivers and lakes. These environments are characterized by low and stable salinity, relatively nutrient-rich conditions, and seasonal hydrological fluctuations, representing the typical growth conditions for *P. australis* in absence of salt stress. | 20 |
| Coastal saltmarsh | Genotypes collected from tidal salt marshes along the eastern coasts of China. Their habitats are defined by frequent tidal inundation, periodic fluctuations in salinity, and waterlogged, anoxic soils, presenting a dynamic saline stress regime influenced by marine processes. | 40 |
| Inland plateau saltmarsh | Genotypes collected from the hyper-saline environments of the Qaidam Basin on the Qinghai-Tibet Plateau. These habitats are characterized by extreme continental climate, high salinity derived from inland salt lakes, strong UV radiation, and arid conditions, representing one of the most stressful natural habitats for *P. australis* in terms of combined salt and drought stress. | 11 |
