# Supplementary material for: Genetic canalization of nutrient resorption in the widespread grass Phragmites australis under salt stress
Source: eLife. 2026 Oct 26;15:RP111792. doi: 10.7554/eLife.111792 (PMC13707377; doi:10.7554/eLife.111792)
Supplement: Peer review history

# Editor report: eLife Assessment

**DOI:** 10.7554/eLife.111792.3.sa0

**Authors:** Sergio Rasmann

**Affiliations:** University of Neuchâtel Switzerland

**Keywords:** Compelling, Important

This **important** study shows that nutrient resorption efficiency in the widespread wetland grass *Phragmites australis* varies with population origin and remains largely stable under experimentally imposed salt stress, supporting limited short-term plasticity and a role for genetic differentiation. The findings suggest that predictions of wetland nutrient cycling under increasing salinization should account for intraspecific variation and phylogeographic composition; the evidence is **compelling**, based on a common-garden experiment involving 110 genotypes, paired control and salinity treatments, and convergent metabolomic, ionomic, and whole-plant evidence confirming substantial physiological stress. The revised manuscript clarifies the partial overlap between ecotype and phylogeographic lineage and appropriately qualifies the modest explanatory contribution of latitude; conclusions remain restricted to one species, one growing season, and a single moderate salinity treatment. An ecotype-specific nitrogen resorption response also indicates that plasticity is not entirely absent and the physiological mechanisms and responses to chronic, stronger, or multigenerational salinity exposure remain unresolved. The study will interest researchers working on plant functional ecology, nutrient cycling, and wetland responses to global change.

# Referee report: Reviewer #1 (Public review)

**DOI:** 10.7554/eLife.111792.3.sa1

Summary:

This study demonstrates that nutrient resorption efficiency (NuRE) in *Phragmites australis* is genetically canalized rather than plastic to salt stress. Using 110 genotypes in a common garden, the authors show that intraspecific variation in NuRE is explained by phylogeographic lineage, ecotype, and latitude, not by effective salinity. Element specific regulatory strategies further reveal how N, P, and K resorption are differentially controlled. At the population level, this is an important study that fundamentally advances our understanding of plant functional trait evolution and its implications for ecosystem nutrient dynamics under global change.

Strengths:

This study is the first to demonstrate genetic determination of a key nutrient conservation trait under effective salt stress in a widespread macrophyte, directly testing the 'plastic acclimation versus inherent conservatism' paradigm in a non-nutrient stress context. The experimental design is rigorous: each genotype was paired across control and salt treatments, and multilevel stress effectiveness (metabolomics, biomass, Na accumulation) was confirmed before evaluating NuRE. The large sample size of a macrophyte and dual classification (phylogeography + ecotype) allow robust disentangling of genetic versus plastic sources of variation.

The analysis comprehensively tests three resorption control hypotheses using appropriate SMA regression, revealing element specific and condition dependent patterns. The latitudinal gradient and variation partitioning provide strong evidence that genetic origin and geographic context outweigh short term plasticity, with important implications for predicting ecosystem nutrient cycling under global change. This study provides a clear empirical demonstration that a key nutrient conservation trait can remain homeostatic under non nutrient stress, and that intraspecific variation is primarily a product of population differentiation rather than short term plasticity.

Weaknesses:

First, the salinity treatment spanned only one growing season. The conclusion of genetic canalization therefore specifically refers to the absence of plasticity to an acute salt shock. Whether long term, multigenerational chronic salinity could act as a selective agent or induce transgenerational plasticity remains an open and interesting question for further research. Likewise, the physiological mechanisms underlying the observed lack of plastic increase in NuRE (for example, phloem loading or senescence gene expression) are not directly resolved, leaving some inference about trade-offs versus true unresponsiveness. These points do not weaken the study's main conclusion. Instead, they suggest productive future directions, such as longer-term field manipulations and targeted molecular investigations.

Second, the test of nutrient limitation control relies on resorbed N:P and N:K ratios as proxies, an established but indirect approach. Direct nutrient addition experiments would provide stronger causal evidence. Also, the metabolomic analysis is used primarily to validate stress effectiveness; deeper integration of specific metabolites with NuRE variation across genotypes could have offered mechanistic insights but was not pursued. Additionally, the potential collinearity between ecotype and phylogeographic lineage among Chinese populations is not quantitatively addressed. None of these considerations undermine the main finding, which is supported by a robust experimental design and widely accepted analytical approaches.

Comments on revised version.

The author carefully revised the parts that might cause confusion.

# Referee report: Reviewer #2 (Public review)

**DOI:** 10.7554/eLife.111792.3.sa2

Summary:

The manuscript shows that nutrient resorption efficiency in *Phragmites australis* is largely genetically canalized rather than plastic under effective salt stress, with variation mainly associated with phylogeographic lineage, ecotype, and latitude.

Strengths:

The common-garden experiment with 110 genotypes, paired control and salt treatments, multi-level stress validation, and element-specific analyses provides compelling support for the central conclusions.

Weaknesses:

The generality is limited by the focus on a single species and a single growing season, and some mechanisms are inferred indirectly.

# Author comment: Author response

**DOI:** 10.7554/eLife.111792.3.sa3

**Authors:** Lele Liu, Wenyi Sheng, Huijia Song, Cui Wang, Na Li, Lele Lin, Yaolin Guo, Ning Du, Weihua Guo

**Affiliations:** Shandong University Qingdao China; Shandong University Qingdao China; National Natural History Museum of China Beijing China; Shandong University Qingdao China; Shandong University Qingdao China; Chinese Academy of Forestry Beijing China; Louisiana State University Agricultural Center Baton Rouge United States; Shandong University Qingdao China; Shandong University Qingdao China

The following is the authors’ response to the original reviews.

The major revisions include:

(1) Conceptual framing and scope: defined canalization in the Introduction and clarified that our conclusions are restricted to limited NuRE plasticity during one growing season under a single moderate salinity treatment.

(2) Methods and classification: clarified the substrate composition and elemental measurements, specified that the ecotype analysis included only Chinese populations, and explained the partial association between ecotype and phylogeographic group.

(3) Interpretation: expanded the discussion of K resorption and inverted nutrient limitation and tempered the interpretation of latitude and the substantial unexplained variation.

(4) Robustness and presentation: added Supplementary Figure S7 showing that carbon standardization did not alter the main conclusions, added significance symbols to Table 1, corrected the unit in Figure 2b, and revised repetitive wording in the Discussion.

**Public Reviews:**

**Reviewer #1 (Public review):**

Weaknesses:

(R1-P1) First, the salinity treatment spanned only one growing season. The conclusion of genetic canalization therefore specifically refers to the absence of plasticity to an acute salt shock. Whether long-term, multigenerational chronic salinity could act as a selective agent or induce transgenerational plasticity remains an open and interesting question for further research. Likewise, the physiological mechanisms underlying the observed lack of plastic increase in NuRE (for example, phloem loading or senescence gene expression) are not directly resolved, leaving some inference about trade-offs versus true unresponsiveness. These points do not weaken the study’s main conclusion. Instead, they suggest productive future directions, such as longer-term field manipulations and targeted molecular investigations.

We agree that our evidence is limited to the absence of NuRE plasticity during one growing season under the imposed salinity treatment and does not resolve chronic or multigenerational responses or their physiological basis. We therefore revised Discussion 4.1 to delimit the canalization inference, identify the proposed mechanisms as untested, and specify the longer-term and mechanistic studies needed to distinguish among them.

Discussion 4.1, fourth paragraph, inserted immediately after the sentence beginning “This discrepancy may stem from differences in the type and duration of stress applied ”.

“Our inference of canalization is therefore limited to the absence of a plastic NuRE response during one growing season under the imposed salinity treatment. Chronic, more severe, or multigenerational salinity exposure may produce acclimatory, epigenetic, or transgenerational responses that cannot be evaluated here. Moreover, although altered phloem loading, disruption of senescence-associated remobilization, and reallocation towards osmotic adjustment are plausible explanations for the observed response, we did not directly measure these mechanisms. Long-term experiments combined with targeted molecular and transport measurements are needed to distinguish among these possibilities.”

(R1-P2) Second, the test of nutrient limitation control relies on resorbed N:P and N:K ratios as proxies, an established but indirect approach. Direct nutrient addition experiments would provide stronger causal evidence. Also, the metabolomic analysis is used primarily to validate stress effectiveness; deeper integration of specific metabolites with NuRE variation across genotypes could have offered mechanistic insights but was not pursued. Additionally, the potential collinearity between ecotype and phylogeographic lineage among Chinese populations is not quantitatively addressed. None of these considerations undermines the main finding, which is supported by a robust experimental design and widely accepted analytical approaches.

We agree and have clarified all three evidential limits. First, the resorbed N: P and N: K analyses are now described as indirect evidence consistent with nutrient-limitation control, not as a causal test; direct nutrient-addition experiments would be required for causal inference. Second, metabolomics is identified as validation of physiological stress rather than a genotype-specific mechanistic analysis. Third, because ecotype and phylogeographic group are partly associated, they were fitted in separate models. The genotype random effect accounts for paired measurements but does not remove confounding between the classification schemes; ecotype differences are therefore interpreted as complementary rather than independent evidence.

Discussion 4.2, third paragraph.

“Within this context, the consistent ‘inverted’ nutrient limitation pattern (i.e., a slope significantly >1 for the relationship between log Resorbed N:P and log Green N:P) provides indirect evidence consistent with nutrient-limitation control at the intraspecific level in *P. australis*, but it cannot establish causal nutrient limitation. Direct factorial nutrient-addition experiments would be required to determine whether the observed resorption patterns are driven by the relative limitation of N, P, or K.”

Discussion 4.1, first paragraph, inserted immediately after the sentence ending “providing a robust foundation to evaluate NuRE responses”.

“In this study, metabolomic profiling was used primarily to confirm that the salinity treatment induced broad physiological stress, rather than to resolve genotype-specific metabolic mechanisms underlying NuRE variation. Integrating metabolite profiles with genotype-level NuRE responses would be a valuable direction for future mechanistic research.”

Methods 2.4.

“Phylogeographic group and ecotype were analysed in separate linear mixed-effects models because ecotype classifications were available only for Chinese populations and were partly associated with phylogeographic structure. Each model included salinity treatment and either phylogeographic group or ecotype as fixed effects, with genotype fitted as a random effect to account for the paired experimental design in which each genotype was exposed to both control and salt conditions.”

Discussion 4.4, first paragraph, inserted immediately after the sentence ending “governed by geographic origin (phylogeographic group and ecotype)”.

“The separate-model approach avoids including the two correlated classification schemes as simultaneous independent predictors, but it does not fully disentangle deep phylogeographic history from recent habitat-associated differentiation. We therefore interpret the ecotype analysis as complementary evidence of habitat-associated differentiation rather than as an effect independent of phylogeographic history.”

**Reviewer #2 (Public review):**

(R2-P1) The experiment covers only one growing season, with salinity applied in June and measurements in December. While the stress is clearly effective, longer-term or multi-year stress might reveal acclimation or epigenetic effects that are not captured. Given the author team’s expertise in parental and transgenerational effects in clonal plants, this limitation is particularly relevant and warrants more thorough discussion in the manuscript.

We agree. This concern overlaps with Reviewer #1’s temporal-scope comment. We have revised Discussion 4.1 to state explicitly that our inference is restricted to the absence of a plastic NuRE response during one growing season. We also acknowledge that chronic or multigenerational exposure could induce acclimatory, epigenetic, or transgenerational responses that were not captured by the present design.

See the full revised text under R1-P1 above.

(R2-P2) The salinity treatment uses a single moderate level of 10 ppt, which does not allow assessment of whether more extreme stress might trigger a plastic response. A dose-response design across a gradient would have provided stronger inference about the threshold at which NuRE canalization might be overcome. Additionally, the ecotype analysis in Figure 4 applies only to Chinese populations, as classification was not available for non-Chinese populations, which should be stated more explicitly in the Results.

We agree with both points. We have revised Discussion 4.1 to acknowledge that the single 10 ppt treatment does not exclude the possibility of a plastic NuRE response at higher salinity or along a broader dose-response gradient. We therefore frame the identification of a possible response threshold as a priority for future experiments.

We have also revised Results 3.2 to state explicitly that the ecotype analysis in Figure 4 included only Chinese populations because ecotype classifications were unavailable for non-Chinese populations.

Discussion 4.1, fourth paragraph, at the same revision point as R1-P1: immediately after the sentence beginning “This discrepancy may stem from differences in the type and duration of stress applied”.

“Because only one moderate salinity level (10 ppt) was tested, our results do not exclude plastic NuRE responses at higher salinity or along a dose-response gradient. Future experiments should determine whether a threshold exists beyond which the apparent stability of NuRE is overcome.”

Results 3.2, second paragraph, inserted immediately after the sentence reporting the ecotype effects and ending “(Figure 4; Figure S5)”.

“It should be noted that the ecotype analysis here was based only on Chinese populations, as ecotype classification was not available for non-Chinese populations.”

(R2-P3) The variation partitioning shows latitude as a significant predictor, but the R^2^ values are relatively low, indicating that much variance remains unexplained. The manuscript should avoid overinterpreting latitude’s explanatory power and more openly acknowledge the role of unmeasured factors. The interpretation of slopes greater than 1 for the resorbed N:P versus green N:P relationship, labeled as “inverted limitation”, also needs further explanation regarding its functional significance.

We agree that the original wording overemphasized latitude. Results 3.4 and Discussion 4.3 now describe latitude as the largest contributor among the measured predictors while emphasizing its modest individual R^2^ and the substantial unexplained variation. We also expanded Discussion 4.2 to explain that slopes greater than 1 indicate disproportionate recovery of P or K relative to N and to present nutrient balance, P conservation, K mobility, and constraints on N remobilization as non-exclusive hypotheses rather than established mechanisms.

Results 3.4, first paragraph, replacing the sentence beginning “Furthermore, variation partitioning analysis indicated that latitude”.

“Variation partitioning indicated that latitude had the largest individual contribution among the measured predictors, but its contribution was modest for N, P, and K resorption (individual R^2^ = 0.092, 0.057, and 0.094, respectively; Table 1). The very small contributions of green-leaf P concentration (R^2^ = 0.006) and stoichiometry (R^2^ < 0.001) further indicate that most variation in P resorption was associated with factors not represented in the present models.”

Discussion 4.3, first paragraph.

“Although latitude explained more variation than the other measured predictors, its individual contribution remained modest. The substantial unexplained variance indicates that additional climatic, edaphic, demographic, or genetic factors also contribute to NuRE variation. We therefore interpret latitude as a significant but limited correlate of NuRE rather than as a dominant determinant.”

Discussion 4.2, first paragraph.

“Functionally, slopes greater than 1 indicate that changes in green-leaf N:P or N: K are accompanied by disproportionate changes in the corresponding resorbed ratio, consistent with relatively greater recovery of P or K than of N across the observed nutrient gradient. This pattern may contribute to maintaining internal N:P:K balance during regrowth. It may also reflect stronger conservation of P, the high mobility of K, or constraints on the remobilization of N retained in structural or metabolic compounds. Because the experiment did not include nutrient additions or direct measurements of remobilization costs, these explanations remain hypotheses.”

**Recommendations for the authors:**

**Reviewer #1 (Recommendations for the authors):**

(R1-R1) Clarify the concept of “canalization” early. Consider adding one sentence in the Introduction or Discussion explicitly defining canalization in this context (i.e., NuRE varies among populations but this variation is genetically determined and shows little plasticity to salinity). This will help readers less familiar with evolutionary biology terminology.

We thank the reviewer for this helpful suggestion. We agree that canalization should be defined when it is first introduced. We have therefore added a sentence to the Introduction, immediately after introducing the evolutionary canalization hypothesis, to clarify how this concept is used in our study.

Introduction, fourth paragraph.

“Here, canalization denotes genetically based differences in NuRE among populations, coupled with limited phenotypic plasticity of this trait under the short-term salinity treatment.”

(R1-R2) Discuss potassium more deeply. In the Discussion (section 4.2 or 4.4), speculate on why K resorption lacks concentration control. Does Na^+^ accumulation functionally substitute for K in osmotic adjustment, thereby decoupling resorption from green leaf K concentration?

We appreciate this suggestion and have expanded Discussion 4.2. We now propose partial functional substitution of K^+^ by accumulated Na^+^ during osmotic adjustment as one possible explanation for the weak coupling between green-leaf K concentration and K resorption. We explicitly frame this explanation as tentative because it was not directly tested in the present experiment.

Discussion 4.2, second paragraph.

“One possible explanation is the partial functional substitution of K^+^ by Na^+^ during osmotic adjustment. Na^+^ can replace part of the nonspecific vacuolar osmotic function of K^+^ in plants and has been reported to become a major osmoticum in *P. australis* from higher-salinity habitats (Wakeel et al., 2011; Zhao et al., 1999). Increased Na^+^ accumulation may therefore reduce reliance on K^+^ for osmotic adjustment, potentially contributing to the weak relationship between green-leaf K concentration and K resorption efficiency observed here.”

Wakeel, A., Farooq, M., Qadir, M., & Schubert, S. (2011). Potassium substitution by sodium in plants. Critical Reviews in Plant Sciences, 30(4), 401–413. https://doi.org/10.1080/07352689.2011.587728

Zhao, K. F., Feng, L. T., & Zhang, S. Q. (1999). Study on the salinity-adaptation physiology in different ecotypes of *Phragmites australis* in the Yellow River Delta of China: Osmotica and their contribution to the osmotic adjustment. Estuarine, Coastal and Shelf Science, 49(Supplement 1), 37–42. https://doi.org/10.1016/S0272-7714(99)80006-7

(R1-R3) Address the potential confounding of ecotype and phylogeography. The dual classification (Figure 3 vs 4) is elegant. However, Chinese ecotypes (freshwater, coastal, inland saltmarsh) may be partially confounded with phylogeographic lineages. A brief sentence explaining how the analytical approach (separate models, random effects) helps separate deep evolutionary history from recent local adaptation would strengthen the interpretation.

We agree. This concern is addressed in detail under R1-P2. We clarified why phylogeographic group and ecotype were fitted in separate models, what the genotype random effect accounts for, and why the ecotype results cannot be interpreted independently of phylogeographic history.

See R1-P2, Locations C1 and C2.

(R1-R4) In section 2.1, specify whether the soil mixture ratio (2 soil: 1 peat moss: 1 river sand) is by volume or by mass.

We thank the reviewer for identifying this ambiguity. The ratio was based on volume, and we have revised Methods 2.1 accordingly.

“The plants were planted in barrels (total volume 25 L; top diameter 32.5 cm, bottom diameter 28.4 cm, height 38.5 cm) containing 20 L of a substrate composed of soil, peat moss, and river sand in a 2:1:1 volume ratio (Figure S1).”

(R1-R5) In the Materials and Methods section, the element potassium (K) is described twice. Specifically, in line 176, the sentence “Besides C, N and P, other eight elements (K, Cu, Zn, Fe, Mn, Mg, Si, Na) were quantified in leaf tissues” should be revised to “Besides C, N, P and K, other seven elements (Cu, Zn, Fe, Mn, Mg, Si, Na) were quantified in leaf tissues” to avoid redundancy.

We thank the reviewer for identifying this redundancy. We have corrected the sentence in Methods 2.2.

“Besides C, N, P and K, seven additional elements (Cu, Zn, Fe, Mn, Mg, Si, and Na) were quantified in leaf tissues.”

(R1-R6) Citations should be formatted and ordered alphabetically or by year of publication.

We thank the reviewer for pointing out this issue. We standardized multi-reference citations, reordered the reference list alphabetically by first-author surname, and verified correspondence between in-text citations and reference entries.

Full-manuscript citation and reference-list audit.

**Reviewer #2 (Recommendations for the authors):**

(R2-R1) In the discussion of the “inverted” nutrient limitation, elaborate on why P and K are recovered more relative to N. Consider whether this reflects a strategy to maintain an optimal N: P: K ratio or arises from higher costs or lower availability of N.

We agree. This issue is addressed in detail under R2-P3, where we explain the meaning of slopes greater than 1 and present the possible functional mechanisms as hypotheses rather than established explanations.

See R2-P3, Location C.

(R2-R2) Expand Table 1 to include confidence intervals or p-values for individual effects. The extremely low R^2^ for concentration and stoichiometry on P resorption should be more explicitly noted as evidence for the dominance of latitude.

We agree and have expanded Table 1 by adding significance symbols (*) to the individual *R*^2^ values. Significance was assessed for the corresponding fixed effects in the full linear mixed-effects models using Type III tests with Satterthwaite’s approximation for degrees of freedom. For P resorption, latitude had the largest individual contribution and was significant (R^2^ = 0.057, *p* = 0.005), whereas the contributions of green-leaf P concentration and stoichiometry were very small and nonsignificant (*R*^2^ = 0.006 and < 0.001, respectively). We revised the Results to emphasize this contrast while acknowledging that latitude explained only a modest proportion of the total variation.

See R2-P3, Locations A and B, for the corresponding Results and Discussion revisions.

(R2-R3) Standardize the abbreviation to “NuRE” throughout, correcting the use of “NRE” in the introduction.

We thank the reviewer for identifying this inconsistency. We standardized the abbreviation to NuRE throughout the manuscript.

(R2-R4) Acknowledge in the discussion that the single moderate salinity level may not have been severe enough to trigger a plastic response, justifying future dose-response experiments.

We agree. This limitation is addressed under R2-P2, where we state that a single 10-ppt treatment cannot exclude plastic responses at higher salinity or along a broader dose-response gradient.

See R2-P2, Location A.

(R2-R5) In the Results section, explicitly state that the ecotype analysis in Figure 4 is based only on Chinese populations, not all 110 genotypes, because ecotype classification was not available for non-Chinese populations.

We agree. This clarification is provided under R2-P2, where Results 3.2 is revised to state that the Figure 4 ecotype analysis includes only Chinese populations.

See R2-P2, Location B.

(R2-R6) Consider adding a supplementary figure comparing raw and carbon-standardized NuRE values to show whether the correction altered main conclusions.

We agree and have added Supplementary Figure S7 comparing raw and carbon-standardized NuRE. The two estimates were strongly correlated for N, P, and K (Pearson’s *r* = 0.997–0.999), and analyses using raw NuRE retained the same conclusions for phylogeographic group, ecotype, salinity, their interactions, and latitude. Thus, carbon standardization slightly shifted the absolute values without altering the main conclusions.

Methods 2.3.

“Raw NuRE was calculated without carbon standardization and compared with carbon-standardized NuRE using Pearson correlations; the main linear mixed-effects analyses were also repeated using raw NuRE.”

Results 3.4, inserted after the existing paragraph reporting the latitude effects and referring to Figure 6 and Table 1.

“Raw and carbon-standardized NuRE were strongly correlated for N, P, and K (Pearson’s r = 0.997–0.999; Figure S7), and analyses using raw NuRE did not alter the conclusions for phylogeographic group, ecotype, salinity, their interactions, or latitude.”

Supplementary Figure S7 legend

“Figure S7 Comparison of raw and carbon-standardized nutrient resorption efficiency (NuRE) for N, P, and K. Points represent genotype-by-treatment observations (n = 206), coloured by treatment. Grey dashed lines indicate the 1:1 relationship, and black lines show ordinary least-squares fits. Pearson’s r and the mean standardized-minus-raw difference (Δmean, percentage points) are shown.”

(R2-R7) In Figure 2b, check the y-axis label; the text reports mg/kg, but the axis shows g/kg, which needs correction.

We thank the reviewer for identifying this unit discrepancy. We corrected the y-axis label in Figure 2b from g/kg to mg/kg so that it matches the units reported in the text.

(R2-R8) In the Discussion, rephrase the sentence “This indicates that NuRE is a conservative trait…” to avoid repetition with the Results summary, for example, “This finding highlights the conservative nature of NuRE.”

We thank the reviewer for this helpful wording suggestion. We have rephrased the sentence to avoid repetition.
